# Molecular mechanisms of neuroendocrine differentiation in prostate cancer progression

**DOI:** 10.1007/s00432-022-04061-7

**Published:** 2022-05-28

**Authors:** Yuchen Xie, Songyi Ning, Jianpeng Hu

**Affiliations:** 1 Affiliated Renmin Hospital of Jiangsu University, Zhenjiang First People’s Hospital, Zhenjiang, 212002 China; 2 Jiangsu University, Zhenjiang, 212013 China

**Keywords:** Androgen deprivation therapy, NEPC, Neuroendocrine differentiation, Molecular mechanism, Prostate cancer

## Abstract

**Background:**

Rapid evolution of the therapeutic management of prostate cancer, especially in in second-generation androgen inhibitors, has increased the opportunity of transformation from prostate cancer (PCa) to neuroendocrine prostate cancer (NEPC). NEPC still lacks effective diagnostic and therapeutic interventions. Researches into the molecular characteristics of neuroendocrine differentiation is undoubtedly crucial to the discovery of new target genes for accurate diagnostic and therapeutic targets.

**Purpose:**

In this review, we focus on the relevant genes and molecular mechanisms that have contributed to the transformation in the progression of PCa and discuss the potential targeted molecule that might improve diagnostic accuracy and therapeutic effectiveness.

**Methods:**

The relevant literatures from PubMed have been reviewed for this article.

**Conclusion:**

Several molecular characteristics influence the progression of neuroendocrine differentiation of prostate cancer which will provide a novel sight for accurate diagnosis and target therapeutic intervention for patients with NEPC.

## Introduction

Androgen deprivation therapy (ADT) has become the standard treatment for patients with PCa, owing to the unique role of the androgen in the growth and progression in prostate cancer (Ren et al. 2013; Wong et al. 2014). Tumors receiving ADT eventually progress to an androgen-resistant state, known as CRPC or more lethal NEPC (Attard et al. 2016). NEPC is an aggressive variant of prostate cancer, characterized by the negative expression of AR and lower level of PSA (Sternberg 2019; Beltran et al. 2014). Patients with NEPC have more frequent distant metastases, more frequent RB1 and TP53 gene loss and express characteristic neuroendocrine markers, such as enolase 2 (eno2), chromogranin A (CHGA), synaptophysin (SYP), etc. (Conteduca et al. 2019; Sagnak et al. 2011).

Emerging researches on molecular mechanisms of neuroendocrine differentiation provide a robust and reliable strategy to understand the molecular features, which identify sensitive biological makers and novel therapeutic targets to improve the lives of the patients with NEPC. Unfortunately, NEPC patients still survive less than one year after diagnosis (Wang et al. 2014). Identification of new targeted genes remains a critical challenge.

### NMYC

The aberrant overexpression and amplifications of N-Myc and Aurka in NEPC contribute to the progression of neuroendocrine differentiation in a synergistic manner (Beltran et al. 2011). Beltran H et al. reported the first paper regarding concurrent AURKA and NMYC gene in NEPC while a potential relationship between AURKA and NMYC has been demonstrated in neuroblastoma (Otto et al. 2009). N-Myc (encoded by MYCN), a transcription factor, is responsible for the growth of brain during embryogenesis and is a key oncogene in oncogenesis of neuroblastoma (Schwab 1993). Medulloblastoma (Thomas et al. 2009), and glioblastoma multiforme (Tateishi et al. 2016). They further validated the results and indicated that N-Myc improve the stabilization of Aurka by inhibiting the interaction with the E3 ubiquitin ligase FBXW7, and blinds to the promoters of target genes including NSE, Syn and AR to regulate their expression, eventually leading to a neuroendocrine phenotype (Beltran et al. 2011). Additionally, the application of Aurora A inhibitor for NEPC models shows great sensitivity and result in the tumor shrinkage and reversal of the phenotype, which identify new therapeutic targets for patients with NEPC (Mosquera et al. 2013).

**Fig. 1:**
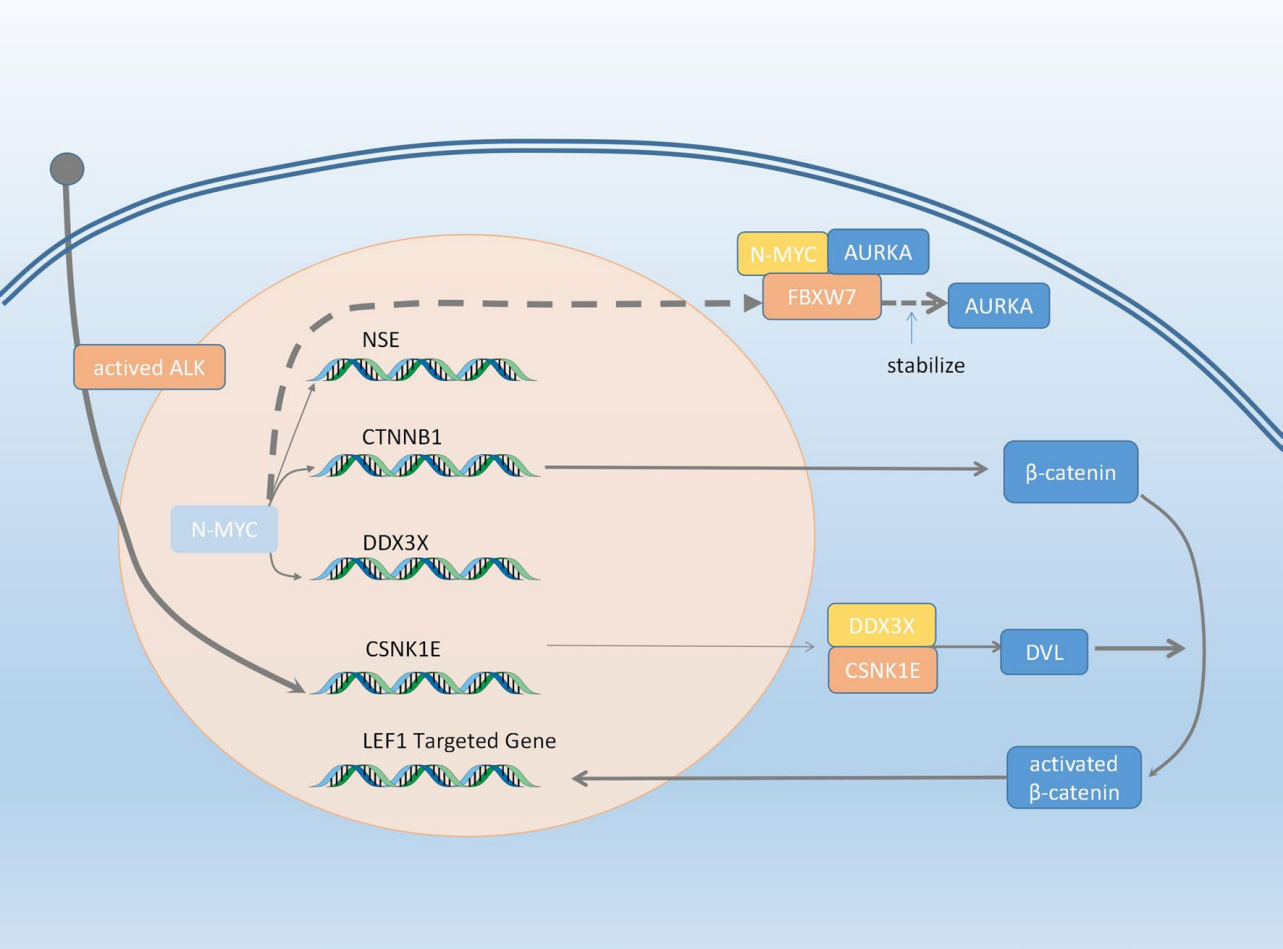
ALK-NMYC and AURKA-NMYC

Concurrent ALK and MYCN gene amplifications contribute to the activation of Wnt/β-catenin signaling pathway in a synergistic manner, leading to the progression of prostate cancer to NEPC. (Unno et al. 2021) (Fig. 1). Anaplastic lymphoma kinase (ALK) is a member of receptor tyrosine kinase family, and the most prevalent alterations of ALK are chromosomal rearrangements leading to fusion genes, which play an oncogenic role in a variety of malignancies, such as non-small cell lung cancer (NSCLC) and anaplastic large cell lymphoma (ALCL) (Du et al. 2018).

Wnt/β-catenin signaling is a canonical pathway in the Wnt family which is dependent on the presence of β-catenin (Wodarz and Nusse 1998).For understanding the underlying mechanism of activation of the canonical Wnt signaling, UnnoK et al. further found that the amplifications of ALK and MYCN gene have a specific impact on the expression of Csnk1e (tyrosine kinase 1) and Ddx3x (DEAD-box RNA helicase) using a lentivirus-mediated gene transfer and tissue recombination model, which contribute to Dvl2 phosphorylation and polymerization in a synergistic manner, leading to activation of β-catenin driving neuroendocrine differentiation(Unno et al. 2021).

### EZH2

**Fig. 2:**
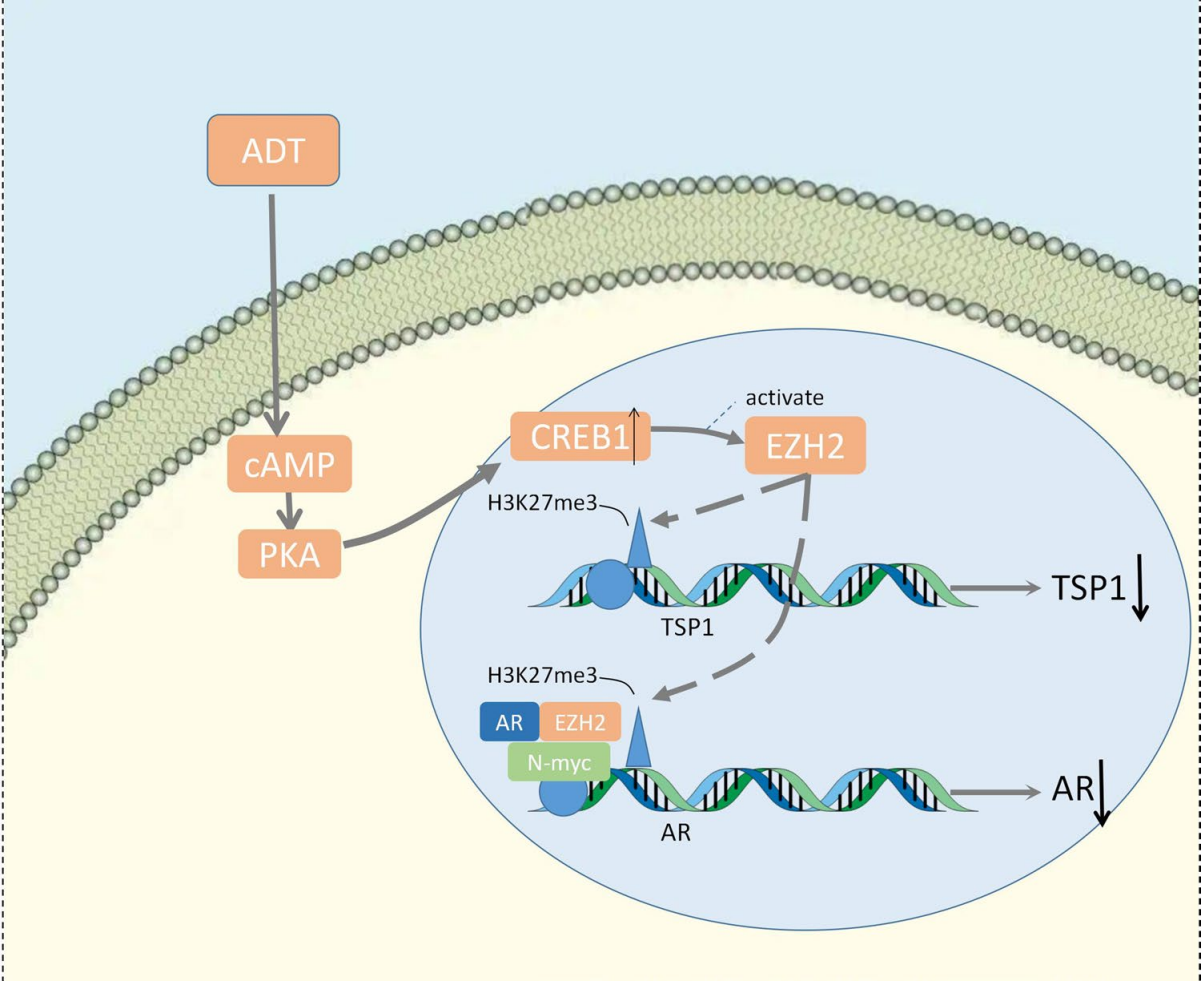
NMYC-EZH2 and CREB1-EZH2-TSP1

The synthesis of N-Myc/AR/EZH2-PRC2 complex is notably dependent on the presence of EZH2, and N-MYC shows a remarkable synergistic effect with EZH2 and AR to inhibit AR signaling by methylation, leading to neuroendocrine transformation of prostate cancer (Dardenne et al. 2016) (Fig. 2). The EZH2 gene is located on chromosome 7q35 (Cardoso et al. 2000) and is a member of the polycomb group gene (PcGs) family. Polycomb repressive complex 2 (PRC2) is a PcG protein core complex that contributes to gene silence through methylation in the promoter of downstream genes (Duan et al. 2020). EZH2, as the catalytic subunit of PRC2, possesses histone methyltransferase activity and have a repressive impact on targeted gene through histone 3 lysine 3 methylation at position 27 (H3K27me3) (Simon and Lange 2008).

Recent researches have found that the expression of EZH2 is significantly upregulated in advanced prostate cancer with distant metastases, supporting the clinical relevance of poor diagnosis the emergence of NEPC in enzalutamide-induced models (Luo et al. 2019; Nadal et al. 2014; Varambally et al. 2002). PCa with ADT elevated cAMP levels and induce the expression of EZH2 by activation of PKA-CREB1 signaling, resulting in the neuroendocrine differentiation and preferentially repressing the expression of TSP1 (Zhang et al. 2018) (Fig. 2). TSP1, as an endogenous angiogenesis inhibitor inhibits proliferation of endothelial cell and induces the apoptosis of endothelial cell (Taraboletti et al. 1990). CREB1 is a 43 kDa transcription factor (TF) (Steven et al. 2020). It functions as a second messenger and activates PKA-CREB signaling which is responsible for overexpression of a variety of downstream genes including the oncogene cyclin D1, leading to tumorigenesis and proliferation of several tumors. (Zhang et al. 2020).

Currently, the molecular features between CREB1 and EZH2 remains controversial. HDAC1 / HDAC2, a downstream target gene of CREB, has attracted attention for its combination with EZH2 in nasopharyngeal carcinoma, suggesting the similar biological role in NEPC (Tong et al. 2012). Collectively, CREB1-EZH2-TSP axis is inevitably involved in the regulation of NE phenotype in prostate cancer progression, which guide a potential determinant for therapeutic strategy in NEPC patients.

### LIN28B

**Fig. 3:**
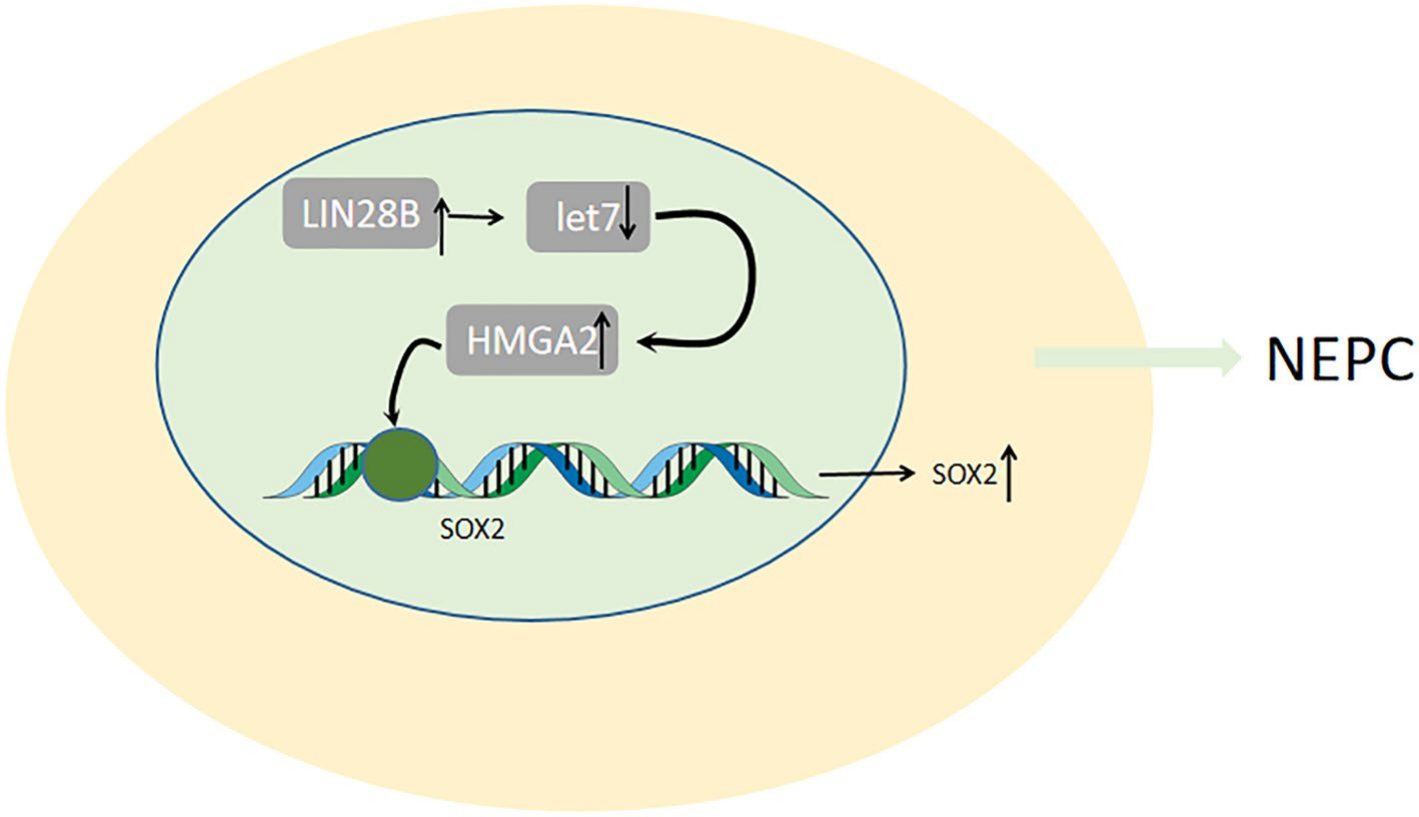
LIN28B/let7/HMGA2/SOX2

Expression of HMGA2 is significantly elevated by LIN28B due to the negative expression of let-7 miRNA. The SOX2 expression is also considerably high with the upregulation of HMGA2, which has been demonstrated to be involved in lineage plasticity and lead to neuroendocrine differentiation by regulating stem cell-like gene networks in PCa (Lovnicki et al. 2020) (Fig. 3). SOX2 belongs to SOX family, located on chromosome 3q26.3-Q27, and is a group of transcription factors (Stevanovic et al. 1994). Recent researches in SOX2 have put emphasis on its key role in stem cell maintenance, lineage fate determination and reprogramming of somatic cells(Sarkar and Hochedlinger 2013).LIN28B, as an RNA-binding protein, selectively inhibit the expression of let-7 miRNA which performs as tumor suppressors and are associated with the downregulation of oncogenes and regulate mitotic pathways including RAS, MYC and HMGA2(Heo et al. 2009; Büssing et al. 2008). Recent improvements provide a novel insight in relationship between LIN28B and various tumor types, including colon cancer(King et al. 2011), ovarian cancer (Lin et al. 2018), liver cancer (Nguyen et al. 2014), neuroblastoma (Chen et al. 2020).

The loss of ESE3/EH downregulated the let-7 microRNAs, an inhibitor of Lin28, leading to the imbalance of Lin28/let-7 and is involved in transformation and Stem-like Phenotype of PCa (Albino et al. 2016). In a word, the Lin28/let-7 axis plays a key role in the induction of transformation of prostate cancer to NEPC which might be conducive to the identification of target gene for existing therapies.

### ONECUT2

The aberrant expression of ONECUT2 in PCa contribute to the upregulation of SMAD3, leading to the activation of hypoxia signaling by HIF1α.The concurrent amplifications of ONECUT2 and hypoxia signaling play an important role in driving neuroendocrine differentiation and the overexpression of NE marker genes (Guo et al. 2019).ONECUT2 is a novel member of the ONECUT family, located on human chromosome 18, and usually functions as a transcription factor, which have fundamental roles during tumorigenesis proliferation, migration and differentiation in hepatocellular carcinoma (Zhang et al. 2015), ovarian cancer(Lu et al. 2018) and lung adenocarcinoma(Ma et al. 2019). The aberrant overexpression of ONECUT2 in CRPC downregulate AR signaling and FOXA1 which are responsible for the neuroendocrine features of CRPC (Rotinen et al. 2018). Conceivably, FOXA1 functions as an inhibitor of neuroendocrine differentiation (Kim et al. 2017).

Cellular plasticity and upregulation of hypoxia response genes in prostate cancer mediated by the overexpression of ONECUT2 are involved in the progression of NE differentiation. It is suggested that treatment directed by inhibition of hypoxia or ONECUT2 might provide a novel insight of therapies to inhibit the progression and occurrence of NEPC.

### PHF8

**Fig. 4:**
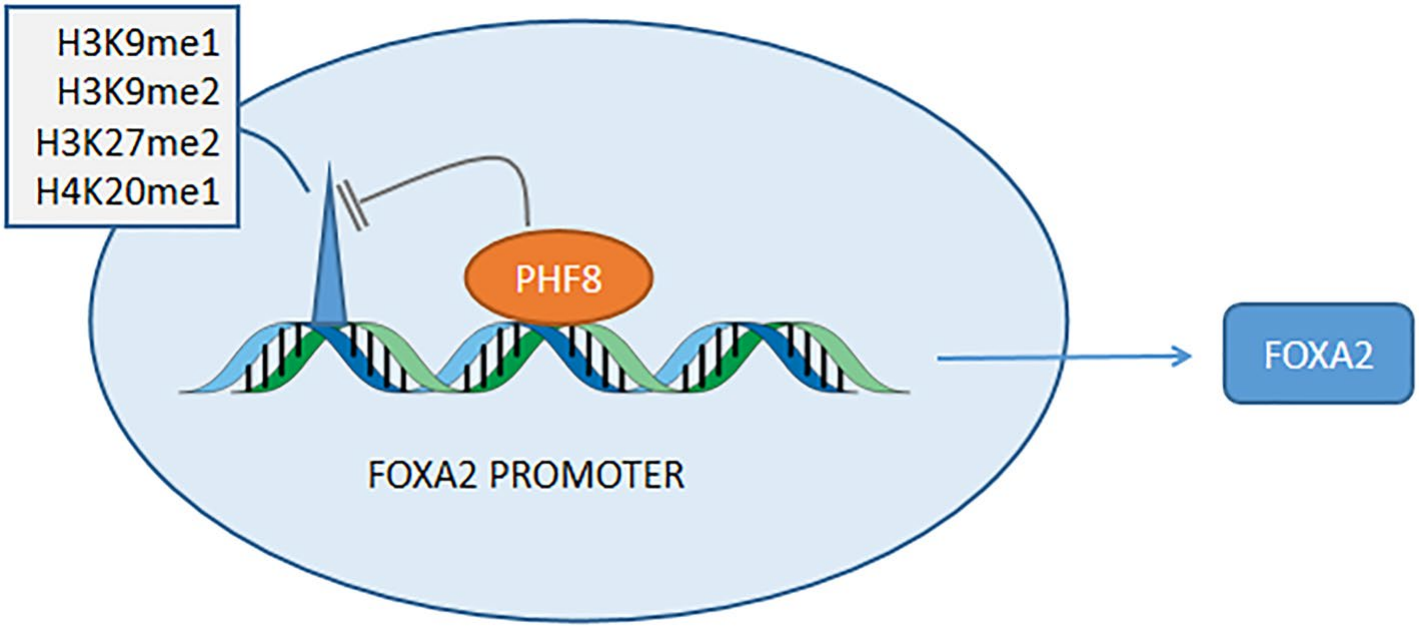
PHF8 upregulates the expression of FOXA2

FOXA2 is incredibly overexpressed due to the removement of repressive methylated proteins in the FOXA2 promoter region by PHF8, which allows for elicitation of aggressive phenotype (Liu et al. 2021) (Fig. 4). The aberrant amplifications of PHF8 are involved in the occurrence, progression and invasion in PCa and intensive researches regarding unique role of PHF8 imply PHF8 as a regulator of neuroendocrine differentiation (Ma et al. 2015; Tong et al. 2016). PHF8 (KDM7B), as a histone demethylase, is responsible for demethylation of H3K9me1/2, H3K27me2 and H4K20me1, allowing transcription of downstream genes (Liu et al. 2010), and is involved in hepatocellular carcinoma (Ye et al. 2019), X-chromosome-linked intellectual disability (XLID) (Chen et al. 2018), melanoma (Moubarak et al. 2022), esophageal cancer (Sun et al. 2013) and so on. FOXA2 is a specific biomarker in NEPC (Park et al. 2017) and can combine with the Siah2-dependent regulation of HIF suggesting the important roles in driving neuroendocrine phenotype (Qi et al. 2010).

Further studies are warranted regarding the relationship between PHF8 and FOXA2 which is still unknown and it might lead to sensitive biological targets for patients with NEPC.

### MUC1-C

MYC-BRN2 is directly or indirectly activated by negative expression of AR axis, in which MUC1-C plays a unique role (Lin et al. 2017; Yasumizu et al. 2020). BRN2, as a neural transcription factor, is negatively correlated with AR activity and identified as an emerging biologic activator of NEPC (Bishop et al. 2017). In addition, MUC1 is a heterodimeric protein that is aberrantly overexpressed in multiple tumors, which contribute to immune evasion in aggressive breast cancer (Maeda et al. 2018; Li et al. 2020a). To date, MUC1-C features the function of EMT, drug resistance, malignant phenotype maintenance (Rajabi and Kufe 2017).

MUC1-C plays a notable role in expression of MYCN, EZH2, and specific biomarkers which have been identified as drivers of NEPC. The molecular features of MYCN and EZH2 in NED have been reported above. Furthermore, repression of the p53 pathway shows a remarkable effect on OCT4, SOX2, KLF4 and MYC pluripotency and drives stemness (Yasumizu et al. 2020). Cell stemness driven by MUC1-C mainly depend on the presence of p53 and SOX2, which regulates lineage plasticity through the LIN28B/HMGA2/SOX axis as described above (Lovnicki et al. 2020). P53 performs as a tumor suppressor and features its alterations in mutations and deletions in a variety of tumors (Khemlina et al. 2015; Akamatsu et al. 2015).

### WLS

**Fig. 5:**
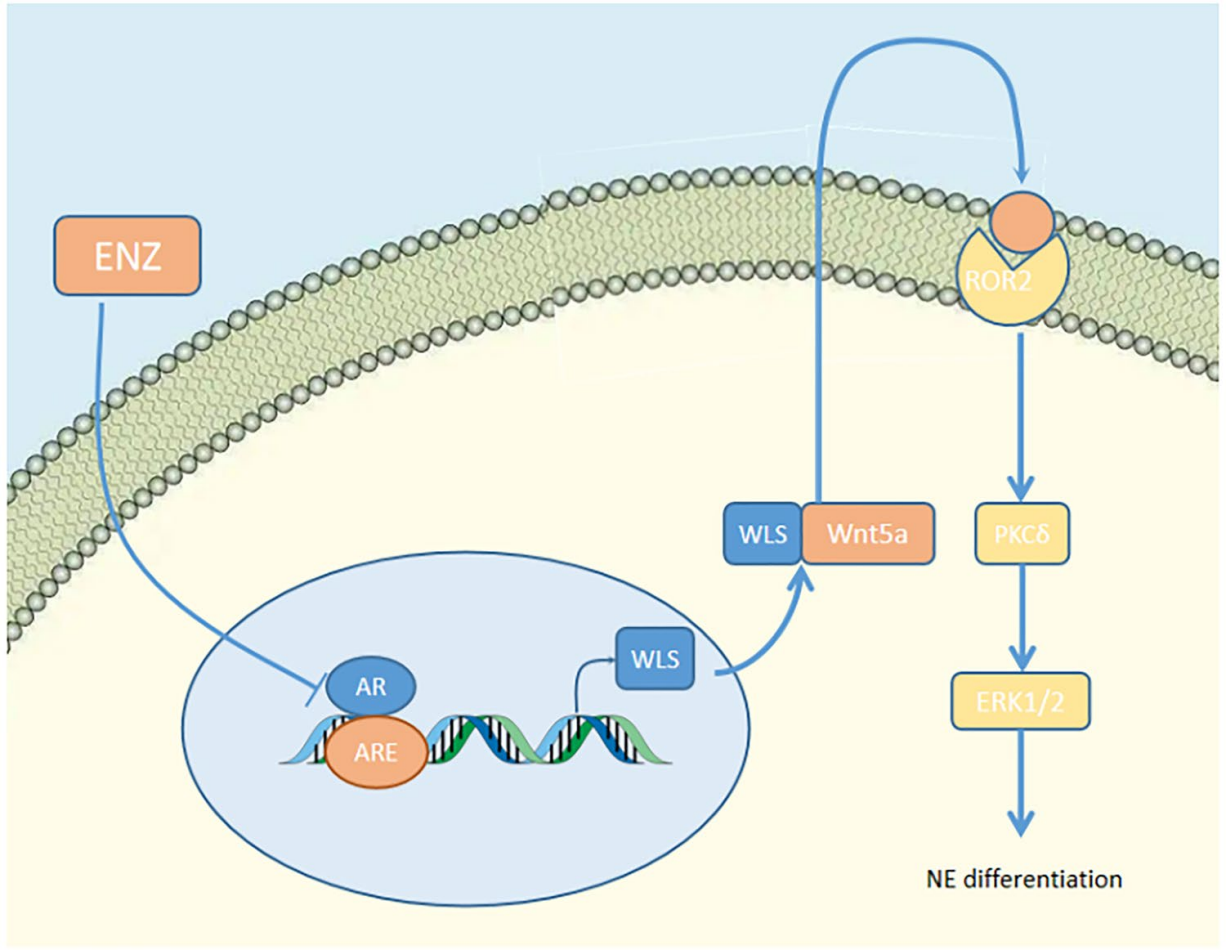
WLS/Wnt

The downregulation of AR by application of androgen potent inhibitor acts as an activator of WLS, leading to the activation of a non-canonical Wnt pathway. Concurrent overexpression of WLS and Wnt5a contribute to downstream ROR2/PKCδ/ERK signaling in a synergistic manner so as to drive neuroendocrine phenotype (Bland et al. 2021) (Fig. 5). ROR2 is an orphan receptor tyrosine kinase and function as a non-canonical Wnt signaling receptor. Wnt5A enhances the chemotaxis and proliferation of leukemia by the activation of ROR1/ROR2 (Yu et al. 2016). Extracellular signal-regulated kinase 1/2 (ERK) belongs to the mitogen-activated protein kinase (MAPK) family and recent improvements emphasize on its effect on signal transmission in signaling cascades (Guo et al. 2020). MAPK/ERK pathway is associated with neuroendocrine phenotype due to the presence of IL-8 (Kim et al. 2017).

WLS (wntless) is a conserved multi-channel endoplasmic reticulum (ER) transmembrane protein.

WLS carrier protein plays a fundamental role in transport and secretion of the Wnt protein after glycosylated and palmitolated in ER into extracellular medium (Bänziger et al. 2006; Smolich et al. 1993). The non-canonical signal consists of Wnt-PCP and Wnt-Ca and features its independence on β-catenin (Komiya and Habas 2008). Wnt4, Wnt5a, and Wnt11 have be reported to be ligands of non-canonical signal (Veeman et al. 2003). Both the canonical pathway and the non-canonical pathway play a vital role in NE differentiation (Unno et al. 2021; Uysal-Onganer et al. 2010). Based on this, inhibition of all WNT signaling may potentially have a unique role in reversing NE transformation, in which WLS is indispensable.

### TROP2

Expression of Trop2 is aberrantly upregulated in NEPC and influence tumor growth, metastasis and neuroendocrine differentiation. Recent studies have identified Trop2 as a driver of aggressive neuroendocrine phenotype, in which elevated PARP1 plays a key role. Furthermore, Trop2-induced NE phenotype can be reversed by PARP1 inhibitors (Hsu et al. 2020). PARP1 is a DNA-dependent ADP- ribosyltransferase has several biologic functions in DNA replication, chromatin remodeling and apoptosis (Ray Chaudhuri and Nussenzweig 2017; Schiewer and Knudsen 2014; Fujimoto et al. 2017). Trophoblast surface antigen 2 (Trop2), also known as tumor-associated calcium signal transducer, is a class of cell surface glycoproteins (Lipinski et al. 1981), elevated levels of Trop2 is closely interrelated to poor prognosis and higher risk of metastasis in various tumors, such as in oral, gastric, thyroid and pancreatic cancer (Fong et al. 2008a, b; Mühlmann et al. 2009; Sun et al. 2021). Additionally, in prostate cancer it consistently enhances tumor growth, migration, metastasis and lineage plasticity (Trerotola et al. 2013).

Elevated levels of Trop2 also contribute to the expression of SOX2 and EZH2 (Hsu et al. 2020), which are associated with lineage plasticity and drug resistance (Li et al. 2020b). To date, although PARP1 is a key factor for TROP2 to act as a NED driver, the underlying mechanism by which TROP2 regulates PARP1 is still unknown. In previous report, c-Myc bind to the promoter of PARP1 and activate its expression to promotes the generation of IPSC and pluripotency maintenance. Similarly, Trop2 may regulate PARP1 through c-Myc to participate in cell reprogramming and NE differentiation (Hsu et al. 2020).

### ZBTB46

**Fig. 6:**
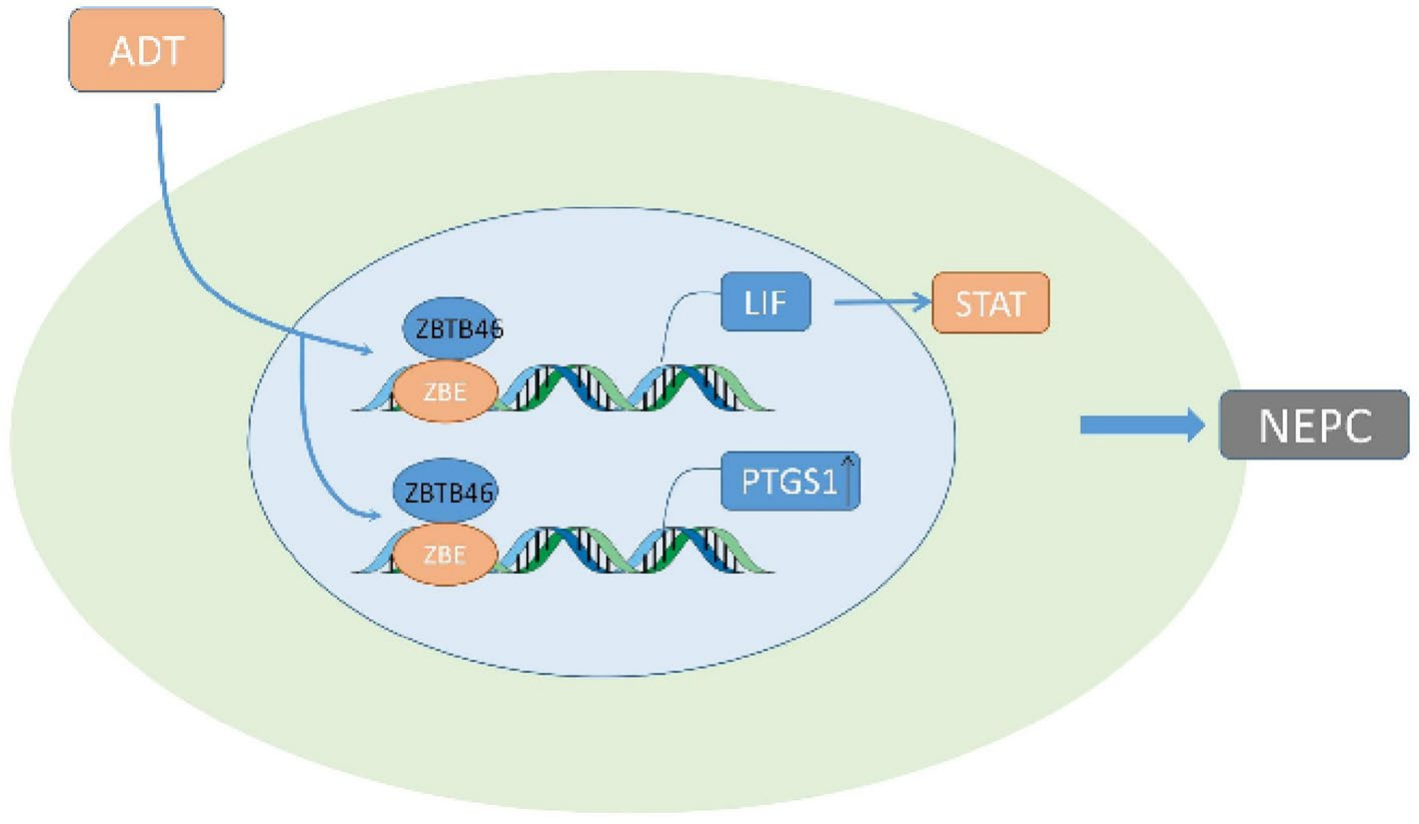
ZBTB46/LIF/STAT and ZBTB46/PTGS1

Downregulation of AR signaling in PCa with ADT leads to elevated levels of LIF and activate LIF-STAT3 pathway to allows for elicitation of aggressive neuroendocrine phenotype. ZBTB46 was involved in the transformation by inactivation of AR signaling (Liu et al. 2019) (Fig. 6). Leukemia inhibitory factor (LIF) is a member of the IL-6 cytokine family. It is a pleiotropic cytokine with a wide range of activities (Nicola and Babon 2015) such as survival, proliferation and metastasis in various tumors through three pathways: JAK/STAT (Stahl et al. 1994), MAP kinase (Williams et al. 2009) and PI (3) kinase (Oh et al. 1998) pathways.

ZBTB46 is a novel tumorigenic factor in prostate cancer, and is negatively correlated with the AR signaling (Chen et al. 2017). It acts as an upstream regulator of Snail and enhance the tumorigenic capacity, progression, invasion and metastasis by regulating the expression of E-cadherin and RKIP in prostate cancer (Beach et al. 2008; Poblete et al. 2014). The inhibition of AR contributes to the upregulation of ZBTB46 and elevated ZBTB46 binds to the promoter of PTGS1 to improve its expression, which is responsible for the malignant transformation and tumors progressing to NEPC (Chen et al. 2019). The androgen-responsive gene SPDEF is prominently down-regulated due to the negative expression of AR signaling during androgen deprivation therapy, resulting in the aberrant elevation of ZBTB46 which is identified as a transcriptional coactivator (Tsai et al. 2018). SPDEF is an E26-specific (ETS) transcription factor (Oettgen et al. 2000), and is considered as a tumor suppressor of PCa (Gu et al. 2007). Downregulation of SPDEF is closely related to the occurrence, growth and metastasis in prostate cancer (Steffan et al. 2016). Prostaglandin G/H synthase 1 (PTGS1) is a physiologically important prostaglandin synthase that plays an important role in the progression of bone diseases, tumors and inflammatory diseases (Smith et al. 2000; Choi et al. 2008; Kargman et al. 1995; Wang et al. 2019).

### EGFR–LIFR-SUCLG2

PCa with ADT induces upregulation of EGFR and acts as a transcriptional regulator that binds to the LIFR promoter to stimulate LIFR expression. The upregulation of LIFR is associated with suclg2. ADT upregulates EGFR-LIFR signaling, activates suclg2, and in turn promotes metabolic changes associated with NE differentiation and an aggressive prostate cancer phenotype (Lin et al. 2020). ADT induced upregulation of the succinate-CoA ligase GDP-forming beta subunit (SUCLG2). Succinate-CoA ligase (SUCL) is a heterodimeric enzyme composed of a Suclg1 α-subunit and a substrate-specific Suclg2 β-subunit, which generates ATP or GTP respectively, this subunit is able to regulate succinate metabolism and NE differentiation in prostate cancer (Kacso et al. 2016).

### Others

Mammalian target of rapamycin (mTOR) acts as an activator of AKT signaling, and aberrant amplifications of constitutively active mTOR leads to NE differentiation and the expression of NSE in LNCaP cells (Kanayama et al. 2017). The level of FOXB2 is significantly upregulated in aggressive PCa which are responsible for NE differentiation by inducing agonistic ligands (mainly WNT7B) to activate the Wnt pathway (Moparthi et al. 2019). It is obvious that the Wnt signaling pathway, both canonical and non-canonical, plays a critical role in inducing the neuroendocrine differentiation process. Heterochromatin protein 1α (HP1α) downregulates the expression of androgen receptor and RE1-silencing transcription factors, and enriches repressive trimethylated histone H3 at Lys9 marks on their respective gene promoters, significantly stimulating NE differentiation and enhanced invasiveness (Ci et al. 2018). Protocadherin-PC (PCDH-PC), encoded on the human Y chromosome, is aberrantly upregulated in prostate cancer tumor cells after hormone deprivation. The upregulation of PCDH-PC results in the upregulation of nuclear β-catenin and induce the NE transformation (Yang et al. 2005; Terry et al. 2006). Interleukin (IL)—6 is associated with progression and differentiation of a variety of tumors (Spiotto and Chung 2000), and STAT3 act as an IL-6-acting mediator to inhibit growth and induce neurite extension and the amplification of NSE expression (Spiotto and Chung 2000). Some cytokines including IL-6 and IL-8 are closely related to the transformation from prostate cancer to NEPC. IL-6 induces NED through SATA3 as described above. IL-8 has direct oncogenicity and can significantly induce cell proliferation, mediate NED and inhibit apoptosis through the STAT3/AKT/NF-κB pathway (Guo et al. 2017).

## Conclusion

NEPC is usually diagnosed at an advanced stage and shows great increase in incidence due to the rapid development of drug resistance. Understanding the driving factors of this kind of highly invasive tumor may provide a theoretical basis for developing the effective treatment strategy. In this review, aimed to better understanding of NEPC progression, we summarize the literature on several genes and pathways that contribute to the development of NEPC. Furthermore, we would like to discuss some others potential and complicate mechanisms in this process to provide some references for further research.

Androgen, as a steroid hormone, maintains the growth and development of PCa by combining with AR. Nevertheless, AR signaling is inhibited in NEPC and how do NEPC survive catches our attention. Further studies are warranted regarding this alternative survival pathway. In the relevant literatures, we found that the upregulation of SREBPs influence the synthesis of enzymes that is critical for lipogenesis and cholesterol synthesis during the progression to androgen independence and the outcome seems to be consistent with our conjecture. Interestingly, CREB interacts with CRTC2 mediating mTOR signaling to regulate the expression of SREBP1 in the liver and PCa with ADT elevated cAMP levels to activate PKA-CREB signaling (Han et al. 2015). On the basis of CREB-SREBPs relationship in liver, we put forward a conjecture that the regulatory mechanisms in NEPC is similar to that in the liver and infer that the upregulation of CREB by elevated cAMP levels in NPEC could interact with CRTC2, a critical mediator of mTOR, to regulate COPII, leading to the upregulation of SREBP-1 and function as an alternative pathway of AR signal. Furthermore, MYC, as an oncogene, activate and cooperate with SREBP-1 to regulate the synthesis of fatty acid and lipogenesis (Gouw et al. 2019). Therefore, we propose the further inference that the upregulation of SREBP-1 by CREB-SREBPs interact with MYC to regulate the lipogenesis and cholesterol synthesis in NEPC and maintain the occurrence and development of NEPC.

The canonical Wnt signal stabilizes β- Catenin and allows its translocation into the nucleus to interact with (TCF/LEF) and coactivators to trigger Wnt target genes and lead to the neuroendocrine differentiation (Ciarlo et al. 2012). During the oviduct development, the canonical WNT cascade increases NR5A2 binding to the CYP11A1 and 3β-HSD gene promoters to facilitate steroidogenesis and regulate oviductal epithelial secretion (Tan et al. 2021). CYP11A1 and 3β-HSD take part in almost all steroidogenic processes and NR5A2 has also been identified as a critical regulator of steroidogenesis (Miller and Auchus 2011). Meanwhile, in the progression of gastrointestinal tumor, the synergy between NR5A21 and β-catenin/TCF4 signaling upregulate the expression of cyclin D1, cyclin E1, and c-Myc which is the downstream targets of the canonical Wnt signaling (Schoonjans et al. 2005). C-Myc is an oncogene that contributes to the genesis of many human cancers and are responsible for proliferation, inflammation, and self-renewing (Dang 2012). On basis of these reports, it can be inferred that the mediation of the canonical Wnt signal might activate the transcription factor NR5A2 and regulate transcriptional control of steroidogenesis by CTNNB1(β-catenin)/NR5A2 signaling in NEPC to replace androgen as a main driver of NEPC growth and progression. Meanwhile, the synergy of β-catenin/TCF4/ NR5A2/c-Myc might play an inevitably role in proliferation and differentiation of NEPC and ALK and MYCN gene amplifications described above might function as the triggering factor to mediate the β-catenin/TCF4/ NR5A2/c-Myc (Unno et al. 2021).

SREBPs, CYP11A1 and 3β-HSD described above are responsible for the formation of steroid hormones, cholesterol and fatty acid and they are inextricably associated with the genes involved in neuroendocrine differentiation like Wnt and CREB, and MYC might to be the bridge between SREBPs, CYP11A1 and 3β-HSD. Therefore, we have every reason to suspect that there is a certain connection between them, although it still remains largely elusive. We believe that the potential connection between them will be made known to the public in the near future to bring hope to patients with NEPC.

## Data Availability

The datasets generated during and/or analysed during the current study are available from the corresponding author on reasonable request.
